# Essential Oils as a Feed Additives: Pharmacokinetics and Potential Toxicity in Monogastric Animals

**DOI:** 10.3390/ani9060352

**Published:** 2019-06-13

**Authors:** Pavel Horky, Sylvie Skalickova, Kristyna Smerkova, Jiri Skladanka

**Affiliations:** 1 Department of Animal Nutrition and Forage Production, Mendel University in Brno, Zemedelska 1, CZ-613 00 Brno, Czech Republic; pavel.horky@mendelu.cz (P.H.); jiri.skladanka@mendelu.cz (J.S.); 2 Department of Chemistry and Biochemistry, Mendel University in Brno, Zemedelska 1, CZ-613 00 Brno, Czech Republic; kristyna.smerkova@mendelu.cz

**Keywords:** phytogenics, natural growth promoters, antibiotic alternatives, genome

## Abstract

**Simple Summary:**

Essential oils are regarded as possible substitutions of antibiotics. Some of them show strong antibacterial effects, and other positive effects in the nutrition of monogastric animals. The article aims to summarise the final state of the art concerning their pharmacokinetics in the organism. Last but not least, great attention is paid to their potential toxic effects.

**Abstract:**

Essential oils (EOs) are now a hot topic in finding modern substitutes for antibiotics. Many studies have shown positive results and confirmed their high antibacterial activity both in vitro and in vivo. Deservedly, there is an attempt to use EOs as a substitute for antibiotics, which are currently limited by legislation in animal breeding. Given the potential of EOs, studies on their fate in the body need to be summarized. The content of EO’s active substances varies depending on growing conditions and consequently on processing and storage. Their content also changes dynamically during the passage through the gastrointestinal tract and their effective concentration can be noticeably diluted at their place of action (small intestine and colon). Based on the solubility of the individual EO’s active substances, they are eliminated from the body at different rates. Despite a strong antimicrobial effect, some oils can be toxic to the body and cause damage to the liver, kidneys, or gastrointestinal tissues. Reproductive toxicity has been reported for *Origanum vulgare* and *Mentha arvensis*. Several publications also address the effect on the genome. It has been observed that EOs can show both genoprotective effects (*Syzygium aromaticum*) and genotoxicity, as is the case of *Cinnamomum camphor*. This review shows that although oils are mainly studied as promising antimicrobials, it is also important to assess animal safety.

## 1. Introduction

Essential oils (EOs) are natural extracts, whose origin is folk medicine. In general, their use is eco-friendly, non-toxic and consistent with nature. Today’s scientific research on EO efficiency is based on different traditional healing systems all over the world. Many preclinical studies have documented antimicrobial, antioxidant, anti-inflammatory, and anticancer activities. Except for the known toxic ingredients, EOs are considered generally regarded as safe (GRAS) in mammals [1,2]. Although many research activities are focused on the toxicity of EOs against insects [3] or aquatic organisms. EOs are unique in that each type differs in the composition of active substances and their concentrations. Currently, approximately 3000 substances in EOs have been identified. The chemical composition of EOs depends on the ambient conditions of plant growth and genetic diversity, which makes it difficult for exploration and commercial exploitation [4,5]. Their representation, combination and quantity results in their properties and behaviour in the organism. Thus, breeding programs for pharmaceutical purposes seem to be a hot topic of research [6,7,8].

Recently, EOs has become an alternative to antibiotics in animal feed. Partly to prevent the antibiotic resistance of microorganisms, but also through the legislative restriction of the frequent use of zinc medication doses [9]. In several in vitro and in vivo studies, it could be found that extracts from rosemary, oregano, dill, cinnamon, eucalyptus, garlic, clove, or thyme were able to modulate ruminal methane emission to various extents, primarily by acting on methanogenic microflora [10]. The mechanisms of action have been summarized in many review articles [11,12,13]. Briefly, these publications describe that EOs components can disrupt the bacterial membrane, damage their metabolic processes or produce reactive oxygen species (ROS) and prevent the synthesis of bacterial toxins [14]. Conversely, some EOs have shown positive effects on microbes [15,16,17]. Moreover, the efficacy of EOs on poultry and swine production have been identified and reviewed [18,19,20]. The cellular protective effects of EOs against drugs or xenobiotics are also described in the literature [21,22,23,24].

The results of some studies demonstrated the efficacy of EOs as feed additives in animal breeding [25]. However, we have found several articles that address their potential toxicity. To provide a comprehensive overview and assess the safety of animals, we bring insights into the issues of their production and pharmacokinetics in mammal organisms.

## 2. Influence of EOs Production on Their Chemical Composition

Due to the EOs complexity, more recent attention has been focused on the choice of extraction procedure that could affect their yield and character. Currently, conventional methods (hydrodistillation, steam distillation, hydro diffusion, and solvent extraction) are alternated by green and sustainable extraction procedures. Benefits include shorter extraction times, lower energy consumption, low solvent usage, and less carbon dioxide emissions [26,27]. Gentle extraction approaches, such as CO_2_ extraction, retain the antioxidant activity of active substances [28]. Nevertheless, successful extraction does not depend on the time of extraction but from an individual approach to the plant material [29]. It has been suggested that levels of the choice of extraction agent also plays a significant role [30]. Comparative studies of the extraction of various oils confirmed the high variability in the composition of the extracted substances that results in their varied effectiveness. The differences in extraction yield are mainly influenced by physicochemical parameters (temperature, time, pH, extraction dynamics), by the technique used or by the inclusion of other steps, such as ohmic heating, the assistance of ultrasound, or ionic liquids [31,32,33,34,35,36,37,38,39].

Factors found to be influencing EOs composition during storage have been explored in several studies. Notably, the exposure to EOs to atmospheric oxygen and UV radiation is one of the major causes of chemical change leading to loss of their efficiency [40,41,42,43]. It has been demonstrated that some phenolic components of EOs are oxidized by contact with reactive oxygen species (ROS) producing very reactive phenoxyl radicals. These types of radical reactions are enhanced by the presence of transition metal ions, such as Fe^2+^, Cu^2+^, Zn^2+^, Mg^2+^, or Mn^2+^ [44,45,46]. In this regard, some studies have shown higher oxidative stability of ethanolic EOs. If storage temperature does not exceed 10 °C, then EOs will maintain their stability for up to eight weeks [47,48]. A growing body of literature has investigated the protection of EOs against oxidation. In this context, non-ionic surfactants, preservatives or stabilizers can be applied [49,50]. The effect has also been demonstrated in the use of gamma radiation with a dose of 20 to 30 kGy. Although results have confirmed an increase in antioxidant activity, there were reduced antibacterial effects due to changing certain active substances caused by gamma radiation [51]. Instead of chemical-physical protection against oxidation, the encapsulation seems to be one of the most perspective methods for preventing the stability changes [52]. Noori et al. used randomly methylated beta-cyclodextrin for encapsulation of *Zingiber officinale* EOs. Comparative tests have confirmed increased antibacterial activity against *E. coli* and *S. aureus,* and antioxidant activity [53].

## 3. EOs Pharmacokinetics in the Organism

An understanding of the pharmacokinetics of EOs in the organism is critical for consideration its effectivity and toxicity. The main obstacle to use EOs as an animal antibiotic is that the active substances enter the intestines at a concentration that is less than inhibitory. Commercial preparations often offer complex mixtures with bonded EOs or the oils are mixed into feed rations. During the digestion process, the individual active substances of EOs are degraded and metabolized. Generally, the kinetics of this process is based on the EOs composition.

EOs are complex mixtures of organic compounds including volatile compounds (such as monoterpene and sesquiterpene hydrocarbons, and their oxygenated derivatives) and of non-volatile residues (such as hydrocarbons, fatty acids, sterols, carotenoids, waxes and flavonoids) [54]. The solubility of the individual active substances has the greatest effect on absorption in the organism. In the gastrointestinal tract, the EOs compounds tend to interact with digested food. As a result, active substances could escape to solubilization and adsorption in the stomach. Moreover, the kinetics rate depends on the activity of digestive enzymes to release the EOs components from the fatty acid bonds [55].

Terpenoids and steroids (carotenoids, phylloxanthins, triterpenoids, and monoterpenes) show lipophilic character. Lipophilic molecules of EOs tend to form micelles, and they are digested in the small intestine together with other lipids. Moreover, their lipophilic character enables them to have easy penetration via the epithelial cell membrane. Thus, the molecule forms are delivered to the small intestine where are released and hydrolysed together with lipids [56,57].

Hydrophilic EOs components (polyphenols, flavones, flavanols, lignans, aromatic acids) are generally bonded to saccharides. These glycosides are metabolised in the small intestine, and their ligands are accessible to enterocytes. Non-absorbed aglycones metabolic pathway passes through the liver, where it is absorbed, and subjected to enzymatic degradation. Besides digestive processes, anthocyanins, isoflavones, tannins metabolism depends on their chemical nature (such as glycosylation) and intestinal microbiota [58,59,60]. Phenolic compounds are known to be unstable in GIT, and they are an easy subject to interaction with other food constituents, or they are hydrolysed during the small intestine passage. [61]. Free hydrophilic molecules are transported into enterocytes via passive diffusion or active transport in the duodenum.

A major proportion of EOs compounds are eliminated by renal excretion, as evidenced by an increase in urine analytes [62]. Non-absorbed and non-metabolized polyphenols leave the body in the faeces [60]. Some studies have reported that the highest concentration of active compounds from EOs is two hours after administration, and after five hours, the substances have been already effectively eliminated from the bloodstream. For example, the half-life of carvacrol, thymol, eugenol, and trans-cinnamaldehyde ranged between 1.84 and 2.05 h, whereas trans-cinnamaldehyde showed the fastest disappearance [63]. Moreover, it was found that the co-existing compounds in *Rhizoma Curcumae* extract could change the pharmacokinetic behaviours [64]. Mason et al. found that residues of the main of oregano and thyme EOs were present up to 13 days in dairy cattle [65,66]. It has been noticed that early absorption could lead to decreasing of antimicrobial effects in the gastrointestinal tract [66]. In this regard, more attention should be paid to the protection of active substances from the undesirable metabolic transformation.

## 4. EOs Toxicity

More recently, literature has emerged that offers contradictory findings of the toxicity of EOs in vitro and in vivo. The discussed health risk includes effects such as respiratory disorders, skin sanitiser, carcinogens, reproductive toxicity, or organ toxicity. In this context, the risk assessment should be taken into account; risk identification, dose-response evaluation, time of exposure, and mechanism of toxicity. The first phase of testing includes in vitro cytotoxicity tests of potentially harmed tissues. Subsequently, the tested substances could be applied in an organism.

EOs have always been used in traditional healing, making many people consider them safe. Although some toxic essences have been deliberately abused for their effects. For example, pennyroyal oil induces abortions and hallucinations, lemon oil is considered as a psychedelic [67,68]. EOs potential to substitute antibiotics are recognized as safe with a long tradition in herbal medicine. In many cases, highly aromatic oils were dosed at low concentrations, which do not cause any damage due to their difficulty to eat. However, poisoning by EOs has been recorded [69]. On the one side, their encapsulation or other kinds of masking their aroma could be risky. On the other side, some studies use the ability of encapsulated EOs to target release, transdermal transfer, enhance permeability effects, or for tissue targeting [70].

Toxicity assessment is complicated by the high variability of the active substance content, which can be up to hundreds in one type of oil [71]. Only a few articles found proposed mechanisms of toxicity. In general, the mechanisms of the cytotoxic effect of EOs are the production of ROS, blockade of sodium channels (e.g., for thymol 150 µM IC50, skeletal muscle cells), cell cycle disruption, mitochondrial damage, DNA aberration, and initialization of NF-kB cascade leading to apoptosis induction [72]. Studies conducted on spermatozoa confirmed that the most sensitive to the dose of EOs is sperm motility, and membrane integrity was minimally affected with a dose of *Melaleuca alternifolia* EOs at a concentration of 0.6 mg/mL [73]. Other oils, such as (*R. officinalis* and *T. capitata*), have also shown similar effects [74,75]. Another toxicological investigation has shown an interesting effect that EOs components in *Salvia officinalis* (α-pinene, camphene, limonene, 1,8-cineole, camphor, borneol, bornyl acetate, α-humulene, viridiflorol, humulene epoxide II, and manool) may exhibit dualistic pharmacological properties. The capability of both neurotransmitter excitatory and inhibition mechanisms in the control of anxiety [76]. Atsumi et al. has shown that the molecular structure plays a role in the toxicity of the active ingredients. Eugenol, the major component of clove EOs, shows much higher cytotoxic effects in its isoform [77]. Moreover, the toxic effects of EOs could be also given by minor ingredients, rather than the most abundant compounds [78]. The comprehensive overview of the studies is given in Table 1.

Although extensive research has been carried out on the antibiotic potential of EOs, there are a few studies elucidated their interaction with nucleic acids. The available studies show that there are two phenomena. At first, genoprotective effect against xenobiotics when EOs have been shown to prevent DNA mutations and aberrations [91,92,93]. Secondly, genotoxicity—mostly for those who are known for their toxicity such as *Syzygium aromaticum, Artemisia absinthium* or *Salvia divinorum*. Model eukaryotic organism *S. cerevisiae* have been analysed on genotoxic effects of *Origanum compactum*, *Artemisia herba alba* and *Cinnamomum camphora* EOs. The primary outcomes of the study suggested that EOs alone caused slight mutations in the cytoplasm, but not in the nucleus. In combination with mutagenic agents (UVB and 8-methoxypsoralen) plus UVA radiation and methylmethane sulfonate, cytoplasmic mutations and mitochondria damage were strictly increased, but nuclear DNA showed no mutagenic changes in combination with genotoxic treatment and EOs [94].

### In Vivo Toxicity

In vivo assays are predominantly conducted to evaluate EOs potential to replace antibiotics both in animals and humans. An overview of publications that monitored the effects of EOs in vivo is given in Table 2. EOs are most commonly associated with hepatotoxicity, nephrotoxicity, changes in the blood vessels, and oxidative stress that occur as a result of acute intoxication. Some EOs have also been associated with reproductive toxicity. It was found that *Origanum vulgare* EOs at a concentration of 27% *v*/*v* affected the size of the genitals. The authors suggested a connection with the possible formation of metabolic syndrome and a decrease in testosterone levels. The highest dose also reduced sperm concentration and induced changes in Leydig cells. Thus, male rat fertility decreased as in a dose-depended manner of the EOs [95]. These results were positively correlated with Shama A. J., who confirmed the contraceptive effects of *Mentha arvensis* at a dose of 10 mg/day/mice treated for 20–60 days. It is also known that some other EOs or their components affect the reproductive system. A new study demonstrated the prenatal toxicity of Verbena EOs. Embryo-fetotoxicity retardation was observed as evidenced by the decrease in foetal weight, head cranium, tail length, and higher incidence in the pre-and post-implantation loss. Some foetal skeleton abnormalities such as incomplete ossification of the skull, sternebrae, and metatarsal bones were observed in foetuses of the 2000 and 3000 mg/kg groups. Flavonoids such as apigenin and luteolin have been identified as major toxicants for the reported prenatal developmental toxicity [96].

In addition to actual toxicity, sub-chronic doses have been monitored. During the 90-day administration of carvacrol/thymol (10:1) at doses of 5, 100 and 200 mg/kg b.w./day, no animals died or showed deviations from the control group. The monitored parameters were overall health state, weight, feed consumption, blood count and histopathological findings. Only glucose levels were decreased, and females had enlarged ovaries in the treated group. The similar results were obtained for *Pinus eldarica* EOs in the dose 125 and 250 mg/kg for 28 days and *Satureja khuzestanica* in the dose for 14 days 0.2–0.6 mL/kg [97,98]. These doses are also found to be tolerable in palatability studies, and also below the LD_50_, which is approximately 2000 mg/kg.

Compared to antibiotics, EOs are tolerable at higher doses. In general, LD_50_ for antibiotics ranges from units up to hundreds of mg/kg for acute administration. The sub-acute toxicity of most antibiotics brings a number of side effects affecting important internal organs. In addition, the discharging of the intestinal microbiota and the resistance of pathogenic microorganisms is still an unsurpassed negative side. Despite the enormous potential of EOs to serve as antibiotic admixtures or feeds, the Guidelines of the Scientific Committee on Food for Safety Assessment (EFSA, 2016) recommend genotoxicity and subchronic studies at the core of tests.

## 5. Conclusions

As a result of the global increase in the demand for antibiotics, EOs are continually being tested for their antimicrobial effects. Several methods for their production have been invented as well as measures to eliminate the extraction of antinutritional or toxic substances contained in natural flavours. Development and research are also growing in the field, for their use in controlling pathogens, or their pharmacological use. The weakness of this research is so far insufficient information about their metabolism in the organism. Some hydrophilic oil components are already absorbed in the stomach, whereas lipophilic oils can pass to the intestine where their site of action is most predicted. The chemical nature of some active substances predisposes them to interactions with other food ingredients and thereby deteriorating their availability. Encapsulation and the use of nanotechnologies seem very promising in this direction. Speculation also leads to their toxicity. Because they are considered GRAS, there is no attempt to investigate their harmful effects on the body, which can cause adverse reactions to organisms. EOs are a considerable issue that needs to be explored in broader contexts and involving more disciplines.

## Figures and Tables

**Table 1:** Cytotoxic effects of EOs (essential oils).

EOs	Main Substances	Cell Line	Dose	Time	Ref.
*Lavandula angustifolia*	linalool 28.9%; linalyl acetate 32.9%	human lymphocytes	0.3 L/mL	24 h	[79]
*Helichrysum italicum*	α-Pinene; camphane; β-Pinene; myrcene; p-cyneme; borneol; thymol				
*Alpinia brevilabris*	α-Pinene 10.1%; β-Pinene 35.3%;	human lung fibroblasts	IC50 90 µg/mL	24 h	[80]
*Alpinia cumingii*	α-Pinene; β-Pinene; ρ-Cymene; α-Terpinene; α-Pinene; α-Cubebene		IC50 70 µg/mL		
*Alpinia elegans*	α-Pinene; β-Pinene; ρ-Cymene; α-Terpinene; α-Pinene; α-Cubebene; 1,8-Cineol		IC50 30 µg/mL		
*Callicarpa micrantha*	β-pinene; caryophyllene epoxide; aristolochene; borneol; linaloo		IC50 85 µg/mL		
*Cinnamomum mercadoi*	Cinnamaldehyde; Camphene; Linalool; α-phellendrene		IC50 215 µg/mL		
*Piper quinqueangulatum*	pinene; khusimene; cadinene		IC50 40 µg/mL		
*Alpinia oxymitra*	Epicatechin; Galloepicatechin	human lung fibroblasts	IC50 10 µg/mL	24 h	[81]
*Boesenbergia rotunda*	2′,4′-dihydroxy-6-methoxychalcone; 5-hydroxy-7-methoxyflavanone; 5,7-dihydroxyflavanone		IC50 20 µg/mL		
*Cinnamomum cambodianum*	Cinnamaldehyde; Camphene; Linalool; α-phellendrene		IC50 110 µg/mL		
*Citrus lucida*	d-limonene		IC50 180 µg/mL		
*Limnophila aromatica*	z-ocimene 39.2%; terpinolene 17.2%; camphor 12.9%		IC50 15 µg/mL		
*Rhodamnia dumetorum*	α-, β-, and γ-eudesmol; α- and β-pinene		IC50 2 µg/mL		
*Sindora siamensis*	α-copaene 41.3%; β-cubebene 15.4%; β-cadinene 7.2%		IC50 6 µg/mL		
*Cinnamodendron dinisii*	1,8-cineole and sabinene.		IC50 30 µg/mL		
*Gallesia integrifolia*	dimethyl trisulfide 15.49%; 2,8-dithianonane; 52.63%; lenthionine; 14.69%	Chinese hamster ovary cell lines	IC50 7 µg/mL	72 h	[82]
*Schinus molle* L.	α-Phellandrene 45.7%; β-phellandrene 13.6%; Hmonene13.4%; α-phellandrene 22.1%, β-phellandrene 10.4%; limonene 9.6%; α-cadinol 5.6%	human lymphocytes	LD50 30.07 μg/mL	72 h	[83]
		human macrophage	LD50 42.07 μg/mL		
*Citrus bergamia*	Limonene 37.2%; Linalyl acetate 30.1%; Linalool 8.8%	mouse fibroblast cells	EC50 0.0023% *v*/*v*	4 h	[84]
*Litsea cubeba*	citral; geraniol; neral.		EC50 0.011% *v*/*v*		
*Citrus X sinensis*	A-pinene; Citronellal; Geranial; Limonene; Linalool; Myrcene; Neral		EC50 0.009% *v*/*v*		
*Cymbopogon citratu*	citral A; citral B; neral		EC50 0.013% *v*/*v*		
*Satureja sahendica Bornm*	Thymol 40%; gamma -terpinene 28%; and rho-cymene 22%	human cancer cell	IC50 15.6 µg/mL	24 h	[85]
*Rosmarinus officinalis*	1,8-cineole; a-pinene; camphor	dermal cell	IC50 5 mL/kg	72 h	[86]
*Piper aduncum*	pinene; khusimene; cadinene	erythrocytes	observed harmful effects 200 μg/mL	24 h	[87]
*Achillea millefolium* L.	thymol 26.47%	macrophages	IC50 22.11 mg/mL	24 h	[88]
*Thymus munbyanus*	thymol 52.0%; gamma-terpinene 11.0%; rho-cymene 8.5%; carvacrol 5.2%	human spermatozoa	500 µg/mL no observed toxicity	30 min	[89]
*Satureja khuzistanica*	Carvacrol 92.87%; limonen 1.2%	cancer cell lines	IC50 125 μg/mL	24 h	[90]

IC50 = half maximal inhibitory concentration; LD50 = median lethal dose; EC50 = half maximal effective concentration.

**Table 2:** Overview of the effects of EOs tested in vivo.

EOs	Main Substances	Organism	Dose	Effects	Ref.
*Syzygium aromaticum*	ugenol (64.74%), caryophyllene (14.36%), 3-Allyl-6-methoxyphenyl acetate (13.28%), 1,4,7, Cycloundecatriene, 1,5,9,9-tetramethy (2.55%).	rats	intraperitoneal injection, 0.125 mg/kg	higher levels of AST, ALT, ALP, decrease of AST hepatotoxicity	[21]
*Commiphora myrrha*	a-pinene, cadinene, limonene, cuminaldehyde, eugenol, m-cresol, heerabolene, acetic acid, formic acid	mice	injection 80 μL	pathological changes on liver and kidney, weight loss	[99]
*Calendula officinalis*	triterpenoid esters, carotenoids flavoxanthin, auroxanthin, lutein, zeaxanthin, flavonol glycosides, triterpene oligoglycosides, oleanane-type triterpene glycosides, saponins, sesquiterpene glucoside	rats	20 mL/kg body weight	higher levels of AST, ALT, ALP	[100]
*Mentha mozaffarianii*	α-Pinene 0.6%; Camphene 0.2%; Sabinene 0.5%; β-Pinene 1.0%; Myrcene 0.3%; Ocymene 0.6%; Limonene 0.4%; 1,8-Cineol 11.7%; Linalool 11.1%; Menthone 1.9%; δ-Terpineol 0.3; Borneol 1.0%; 4-Terpineol 0.2%; α-Terpineol 3.4%; Pulegone 0.3%; Piperitone 51.0% Thymol 1.0%; Piperitenone 8.6%; Piperitenone oxide 2.3%; Trans-Jasmone 1.9%; β-Caryophyllene 0.8%	rats	2000 mg/kg diet	higher level of glucose, cholesterol, ALT, AST, ALP, and TSH; tissue damage of liver, kidney, stomach	[101]
*Trachyspermum ammi*	Thymol 58,9%; p-cymene 24.02%; γ-terpinene 13.77 %; β-pinene 1.90%	mice	7% acute dermal irritation	defined erythema	[102]
*Boenninghausenia albiflora*	propyl ether 22%; linalool 22%; cinnamaldehyde 15%; cinnamyl alcohol 5%	rats	400 mg/kg diet	changes in the clinical picture (RBC, MCV, triglycerides, HDL, LDL, urea, and sodium)	[103]
*Cuminum cyminum L.*	Cuminaldehyde; cymene; terpenoids	rats	1000 mg/kg diet	increase of serum levels of ALT	[104]
*Satureja khuzestanica*	Carvacrol 11%; Thymol 28.2%; γ-terpinene 16%; ρ-cymene 19.6%; β-pinene 4.5%; Sabinene 4.4%	mice	injection 1.79 mL/ kg body weight	death	[98]
*Artemisia vulgaris L.*	Camphor; 1,8-cineole	rats	10.3–23.1 mg/kg body weight	anaemia	[105]
*Aquilaria crassna*	sabinene; linalyl acetate; anisaldehyde; perillaldehyde; 3-carvomenthenol; 3-carvomenthenone; bornyl acetate; p-mentha-1,3-dien-7-ol; cuminic acid; p-mentha-1,3-dien-7-av	mice	2000 mg/kg/day orally	weight loss	[106]
*Salvia officinalis*	Camphor 25%; 1,8-cineole 7.5%; α-tujone 22.2%	rats	30 mg/kg body weight	induced hepatotoxicity, lipid peroxidation	[107]
*Curcuma longa*	cinnamic acid; 5 malonyl-CoA; p-coumaric acid	rats	5000 mg/kg body weight	No changes in the monitored parameters	[108]
*Piper vicosanum*	monoterpenoids 56.0–62.6%; limonene 40.0–45.5%; 1,8-cineole 10.4–15.0%	rats	2 g/kg body weight		[109]
*Lavandula angustifolia*	Linalool, Camphor and 1,8- cineole	mice, rabbits	2000 mg/kg diet		[110]
*Cinnamomum zeylanicum*	Cinnamaldehyde, Camphene, Linalool and α-phellendrene	mice	1.52 mL/kg body weight		[111]
*Origanum vulgare*	Carvacrol 80%; Thymol 64%; γ-terpinene 52%; ρ-cymene 52%	rats	200 mg/ kg body weight		[112]
*Satureja khuzestanica*	Carvacrol 11%; Thymol 28.2%; γ-terpinene 16%; ρ-cymene 19.6%; β-pinene 4.5%; Sabinene 4.4%	mice	0.2, 0.4 and 0.8 mL/ kg diet		[98]
*Piper glabratum*	pinene 12.0%; khusimene 12.1%; cadinene 13.2%	mice	5000 mg/kg/body weight		[113]
*Lavandula stoechas*	Linalool, Camphor and 1,8-cineole	rats	200 mg / kg body weight		[114]
*Ocimum sanctum L.*	Oleanolic acid, Ursolic acid, Rosmarinic acid, Eugenol, Carvacrol, β-caryophyllene and β-elemene.	mice	LD50 4571.43 µL/kg	death	[115]
*Mentha mozaffarianii*	Linalool 51.8%; Epoxyocimene 19.3%; Sesquiphellandrene 9.4%; Cadinene 4.0%	rats	LD50 greater than 2000 mg/kg	increases blood glucose, cholesterol, ALT, AST, ALP, and TSH	[101]
*Lavandula stoechas*	1,8-cineole; lavandulol; necrodane	rats	200 mg/kg	No changes in the monitored parameters	[114]
*Origanum vulgare*	Carvacrol 80%; Thymol 64%; γ-terpinene 52%; ρ-cymene 52%	rats	3% diet	No changes in the monitored parameters (spermatozoa)	[95]
*Origanum vulgare*	Carvacrol 80%; Thymol 64%; γ-terpinene 52%; ρ-cymene 52%	rats	200 mg/kg b.w.	Data revealed no mortality and no treatment-related adverse effects of the EOs in food/water consumption, body weight, haematology, biochemistry, necropsy, organ weight and histopathology.	[112]
*Ocimum gratissimum*	Oleanolic acid; Ursolic acid; Rosmarinic acid; Eugenol; Carvacrol; β-caryophyllene; β-elemene	rats	1500 mg/kg body weight	No changes in the monitored parameters (functional damages to stomach and liver)	[116]
*Thymbra capitata (L.)*	1,8-cineole 19.60%; Camphor 17%; α-pinene 15.12%; Borneol 8.17%; Verbenone 9.55%	boars	0.6 mg/mL	No changes in the monitored parameters (spermatozoa)	[74]
*Eucalyptus staigeriana*	Cineole 46.8%; α-pinene 28.9%; d-limonene 4.9%	rats	LD50 3.495.9 mg/mL	death	[117]
*Eucalyptus Eucalyptus*	Cineole 6.2%; α-pinene 8.3%; ρ-cymene 28.6%; Cryptone 17.8%; Cuminaldehyde 6.5%	rats	LD50 2.334 mg/kg b.w.	death	[118]
*Eugenia caryophyllus*	eugenol; isoeugenol; eugenone; β-caryophyllene	rats	LD50 3.597 mg/kg b.w.	death	
*Pinus eldarica*	Thymol 78.8%; karvarol 6,2%	rats	LD50 higher than 22.5 mL/kg b.w.	No changes in the monitored parameters	[119]
*Verbena officinalis*	1-octen-3-ol 30.76%; Verbenone 20.49%	pregnant female rats	3000 mg/kg diet	asymmetrical distribution of implantation sites and embryos	[96]
*Verbena litoralis Kunth*	Epicatechin; Galloepicatechin; Cadinene	rats	400 mg/kg diet	only increase in AST	[120]
*Lantana camara*	bicyclogermacrene 19.4%; isocaryophyllene; 16.7%; valencene 12.9%; germacrene D 12.3%	guinea pigs	24 mg/kg b.w.	decrease in weekly body weights, haematology, liver and kidney marker enzymes (ALT, AST, ALP and creatinine)	[121]

AST = aspartate transaminase; ALT = alanine transaminase; ALP = alkaline phosphatase; TSH = hydroid stimulating hormone; RBC = red blood cell count; MCV = mean corpuscular volume; HDL = high-density lipoprotein; LDL = low-density lipoprotein.

## References

[1] Qiao Z.Y., Dai S.N., Zhang Q.J., Yang W.G., Chen J. (2018). Predicting cytotoxicity of essential oils from traditional chinese medicine with machine learning technique. Basic Clin. Pharmacol. Toxicol..

[2] Horky P., Skladanka J., Nevrkla P., Slama P. (2016). Effect of diet supplemented with antioxidants (selenium, copper, vitamins E and C) on antioxidant status and ejaculate quality of breeding boars. Ann. Anim. Sci..

[3] Benelli G., Pavela R., Petrelli R., Cappellacci L., Canale A., Senthil-Nathan S., Maggi F. (2018). Not just popular spices! Essential oils from cuminum cyminum and pimpinella anisum are toxic to insect pests and vectors without affecting non-target invertebrates. Ind. Crop Prod..

[4] Nazem V., Sabzalian M.R., Saeidi G., Rahimmalek M. (2019). Essential oil yield and composition and secondary metabolites in self- and open-pollinated populations of mint (*Mentha* spp.). Ind. Crop Prod..

[5] Tammar S., Salem N., Rebey I.B., Sriti J., Hammami M., Khammassi S., Marzouk B., Ksouri R., Msaada K. (2019). Regional effect on essential oil composition and antimicrobial activity of *Thymus capitatus* L.. J. Essent. Oil Res..

[6] Ricroch A.E., Henard-Damave M.C. (2016). Next biotech plants: New traits, crops, developers and technologies for addressing global challenges. Crit. Rev. Biotechnol..

[7] Bechtold U. (2018). Plant life in extreme environments: How do you improve drought tolerance?. Front. Plant Sci..

[8] Glass S., Fanzo J. (2017). Genetic modification technology for nutrition and improving diets: An ethical perspective. Curr. Opin. Biotechnol..

[9] Horky P., Skalickova S., Urbankova L., Baholet D., Kociova S., Bytesnikova Z., Kabourkova E., Lackova Z., Cernei N., Gagic M. (2019). Zincphosphate-based nanoparticles as a novel antibacterial agent: In vivo study on rats after dietary exposure. J. Anim. Sci. Biotechnol..

[10] Cobellis G., Trabalza-Marinucci M., Yu Z.T. (2016). Critical evaluation of essential oils as rumen modifiers in ruminant nutrition: A review. Sci. Total Environ..

[11] Reyes-Jurado F., Franco-Vega A., Ramirez-Corona N., Palou E., Lopez-Malo A. (2015). Essential oils: Antimicrobial activities, extraction methods, and their modeling. Food Eng. Rev..

[12] Omonijo F.A., Ni L.J., Gong J., Wang Q., Lahaye L., Yang C.B. (2018). Essential oils as alternatives to antibiotics in swine production. Anim. Nutr..

[13] Rao J.J., Chen B.C., McClements D.J. (2019). Improving the efficacy of essential oils as antimicrobials in foods: Mechanisms of action. Ann. Rev. Food Sci. Technol..

[14] Tohidi B., Rahimmalek M., Arzani A. (2017). Essential oil composition, total phenolic, flavonoid contents, and antioxidant activity of *Thymus species* collected from different regions of Iran. Food Chem..

[15] Li Y., Fu X.F., Ma X., Geng S.J., Jiang X.M., Huang Q.C., Hu C.H., Han X.Y. (2018). Intestinal microbiome-metabolome responses to essential oils in piglets. Front. Microbiol..

[16] Li D.H., Wu H.J., Dou H.T., Guo L., Huang W. (2018). Microcapsule of sweet orange essential oil changes gut microbiota in diet-induced obese rats. Biochem. Biophys. Res. Commun..

[17] Asli M.Y., Khorshidian N., Mortazavian A.M., Hosseini H. (2017). A review on the impact of herbal extracts and essential oils on viability of probiotics in fermented milks. Curr. Nutr. Food Sci..

[18] Zhai H.X., Liu H., Wang S.K., Wu J.L., Kluenter A.M. (2018). Potential of essential oils for poultry and pigs. Anim. Nutr..

[19] Horky P., Tmejova K., Kensova R., Cernei N., Kudr J., Ruttkay-Nedecky B., Sapakova E., Adam V., Kizek R. (2015). Effect of heat stress on the antioxidant activity of boar ejaculate revealed by spectroscopic and electrochemical methods. Int. J. Electrochem. Sci..

[20] Patra A.K., Amasheh S., Aschenbach J.R. (2018). Modulation of gastrointestinal barrier and nutrient transport function in farm animals by natural plant bioactive compounds—A comprehensive review. Crit. Rev. Food Sci. Nutr..

[21] Abd Al-Azem D., Al-Derawi K.H., Al-Saadi S.A.A.M. (2019). The protective effects of *Syzygium aromaticum* essential oil extract against methotrexate induced hepatic and renal toxicity in rats. J. Pure Appl. Microbiol..

[22] Bellassoued K., Ghrab F., Hamed H., Kallel R., van Pelt J., Lahyani A., Ayadi F.M., El Feki A. (2019). Protective effect of essential oil of *Cinnamomum verum* bark on hepatic and renal toxicity induced by carbon tetrachloride in rats. Appl. Physiol. Nutr. Metab..

[23] Bouzenna H., Samout N., Dhibi S., Mbarki S., Akermi S., Khdhiri A., Elfeki A., Hfaiedh N. (2019). Protective effect of essential oil from *Citrus limon* against aspirin-induced toxicity in rats. Hum. Exp. Toxicol..

[24] Rao Z., Xu F., Wen T., Wang F., Sang W., Zeng N. (2018). Protective effects of essential oils from *Rimulus cinnamon* on endotoxin poisoning mice. Biomed. Pharmacother..

[25] Cheng C.S., Xia M., Zhang X.M., Wang C., Jiang S.W., Peng J. (2018). Supplementing oregano essential oil in a reduced-protein diet improves growth performance and nutrient digestibility by modulating intestinal bacteria, intestinal morphology, and antioxidative capacity of growing-finishing pigs. Animals.

[26] Aziz Z.A.A., Ahmad A., Setapar S.H.M., Karakucuk A., Azim M.M., Lokhat D., Rafatullah M., Ganash M., Kamal M.A., Ashraf G.M. (2018). Essential oils: Extraction techniques, pharmaceutical and therapeutic potential—A review. Curr. Drug Metab..

[27] Giacometti J., Kovacevic D.B., Putnik P., Gabric D., Bilusic T., Kresic G., Stulic V., Barba F.J., Chemat F., Barbosa-Canovas G. (2018). Extraction of bioactive compounds and essential oils from Mediterranean herbs by conventional and green innovative techniques: A review. Food Res. Int..

[28] Topal U., Sasaki M., Goto M., Otles S. (2008). Chemical compositions and antioxidant properties of essential oils from nine species of Turkish plants obtained by supercritical carbon dioxide extraction and steam distillation. Int. J. Food Sci. Nutr..

[29] Bozovic M., Navarra A., Garzoli S., Pepi F., Ragno R. (2017). Esential oils extraction: A 24-hour steam distillation systematic methodology. Nat. Prod. Res..

[30] Ajila C., Brar K., Verma M., Tyagi R.D., Godbout S., Valero J.R. (2010). Extraction and analysis of Polyphenols: Recent trends. Crit. Rev. Biotechnol..

[31] Tavakolpour Y., Moosavi-Nasab M., Niakousari M., Haghighi-Manesh S., Hashemi S.M.B., Khaneghah A.M. (2017). Comparison of four extraction methods for essential oil from *Thymus daenensis* subsp. *lancifoliusand* chemical analysis of extracted essential oil. J. Food Process. Preserv..

[32] Hashemi S.M.B., Khaneghah A.M., Koubaa M., Barba F.J., Abedi E., Niakousari M., Tavakoli J. (2018). Extraction of essential oil from *Aloysia citriodora* palau leaves using continuous and pulsed ultrasound: Kinetics, antioxidant activity and antimicrobial properties. Process Biochem..

[33] Asl R.M.Z., Niakousari M., Gahruie H.H., Saharkhiz M.J., Khaneghah A.M. (2018). Study of two-stage ohmic hydro-extraction of essential oil from *Artemisia aucheri* Boiss.: Antioxidant and antimicrobial characteristics. Food Res. Int..

[34] Golmakani M.T., Moayyedi M. (2015). Comparison of heat and mass transfer of different microwave-assisted extraction methods of essential oil from *Citrus limon* (Lisbon variety) peel. Food Sci. Nutr..

[35] Jaimand K., Rezaee M.B., Homami S. (2018). Comparison extraction methods of essential oils of *Rosmarinus officinalis* L. In Iran by microwave assisted water distillation; water distillation and steam distillation. J. Med. Plants By-Prod. JMPB.

[36] Konoz E., Hajikhani N., Abbasi A. (2017). Comparison of two methods for extraction of dill essential oil by gas chromatography-mass spectrometry coupled with chemometric resolution techniques. Int. J. Food Prop..

[37] Nekoei M., Mohammadhosseini M. (2017). Chemical composition of the essential oils and volatiles of *Salvia leriifolia* by three different extraction methods prior to gas chromatographic-mass spectrometric determination: Comparison of HD with SFME and HS-SPME. J. Essent. Oil Bear. Plants.

[38] Samejo M.Q., Memon S., Bhanger M.I., Khan K.M. (2013). Comparison of chemical composition of *Aerva javanica* seed essential oils obtained by different extraction methods. Pak. J. Pharm. Sci..

[39] Lanari D., Marcotullio M.C., Neri A. (2018). A design of experiment approach for ionic liquid-based extraction of toxic components-minimized essential oil from *Myristica fragrans* Houtt. Fruits. Molecules.

[40] Jia B., Xu L.X., Guan W.Q., Lin Q., Brennan C., Yan R.X., Zhao H. (2019). Effect of citronella essential oil fumigation on sprout suppression and quality of potato tubers during storage. Food Chem..

[41] Perna A., Simonetti A., Gambacorta E. (2019). Phenolic content and antioxidant activity of donkey milk kefir fortified with sulla honey and rosemary essential oil during refrigerated storage. Int. J. Dairy Technol..

[42] Gottschalk P., Brodesser B., Poncelet D., Jaeger H., Rennhofer H., Cole S. (2019). Impact of storage on the physico-chemical properties of microparticles comprising a hydrogenated vegetable oil matrix and different essential oil concentrations. J. Microencapsul..

[43] Sivakumar D., Bautista-Banos S. (2014). A review on the use of essential oils for postharvest decay control and maintenance of fruit quality during storage. Crop Prot..

[44] Ami A.S., Bhat S.H., Hanif S., Hadi S.M. (2006). Plant polyphenols mobilize endogenous copper in human peripheral lymphocytes leading to oxidative DNA breakage: A putative mechanism for anticancer properties. FEBS Lett..

[45] Bakkali F., Averbeck S., Averbeck D., Waomar M. (2008). Biological effects of essential oils—A review. Food Chem. Toxicol..

[46] Huang H.C., Wang H.F., Yih K.H., Chang L.Z., Chang T.M. (2012). The dual antimelanogenic and antioxidant activities of the essential oil extracted from the leaves of *Acorus macrospadiceus* (Yamamoto) F. N. Wei et Y. K. Li. Evid.-Based Complement. Altern. Med..

[47] Naeem A., Abbas T., Ali T.M., Hasnain A. (2018). Effect of storage on oxidation stability of essential oils derived from culinary herbs and spices. J. Food Meas. Charact..

[48] Turek C., Stintzing F.C. (2013). Stability of essential oils: A review. Compr. Rev. Food Sci. Food Saf..

[49] Chadli S., Mourad L., El-Hadj A., Aissou M., Boudjema F. (2019). Impact of tween 60 on physicochemical properties and stability of *Pistacia lentiscus* fruit oil-in-water emulsion at a semi-low temperature. J. Dispers. Sci. Technol..

[50] Olmedo R., Ribotta P., Grosso N.R. (2019). Decrease of chemical and volatile oxidation indicators using oregano essential oil combined with BHT in sunflower oil under accelerated storage conditions. J. Food Sci. Technol..

[51] Alloun K., Benchabane O., Hazzit M., Mouhouche F., Baaliouamer A., Chikhoune A., Benchabane A. (2019). Effect of gamma ray irradiation on chemical composition, antioxidant, antimicrobial, and insecticidal activities of *Thymus pallescens* essential oil. Acta Chromatogr..

[52] Kfoury M., Auezova L., Greige-Gerges H., Fourmentin S. (2019). Encapsulation in cyclodextrins to widen the applications of essential oils. Environ. Chem. Lett..

[53] Noori S., Zeynali F., Almasi H. (2018). Antimicrobial and antioxidant efficiency of nanoemulsion-based edible coating containing ginger (*Zingiber officinale*) essential oil and its effect on safety and quality attributes of chicken breast fillets. Food Control.

[54] De Groot A.C., Schmidt E. (2016). Essential oils, part III: Chemical composition. Dermatitis.

[55] Rubio L., Macia A., Motilva M.J. (2014). Impact of various factors on pharmacokinetics of bioactive polyphenols: An overview. Curr. Drug Metab..

[56] Rodriguez-Concepcion M., Avalos J., Bonet M.L., Boronat A., Gomez-Gomez L., Hornero-Mendez D., Limon M.C., Melendez-Martinez A.J., Olmedilla-Alonso B., Palou A. (2018). A global perspective on carotenoids: Metabolism, biotechnology, and benefits for nutrition and health. Prog. Lipid Res..

[57] Papada E., Gioxari A., Brieudes V., Amerikanou C., Halabalaki M., Skaltsounis A.L., Smyrnioudis I., Kaliora A.C. (2018). Bioavailability of terpenes and postprandial effect on human antioxidant potential. An open-label study in healthy subjects. Mol. Nutr. Food Res..

[58] Liu Y.X., Zhang D., Wu Y.P., Wang D., Wei Y., Wu J.L., Ji B.P. (2014). Stability and absorption of anthocyanins from blueberries subjected to a simulated digestion process. Int. J. Food Sci. Nutr..

[59] Williamson G., Kay C.D., Crozier A. (2018). The bioavailability, transport, and bioactivity of dietary flavonoids: A review from a historical perspective. Compr. Rev. Food Sci. Food Saf..

[60] Lin S.L., Wang Z.Y., Lam K.L., Zeng S.X., Tan B.K., Hu J.M. (2019). Role of intestinal microecology in the regulation of energy metabolism by dietary polyphenols and their metabolites. Food Nutr. Res..

[61] Pinto J., Spinola V., Llorent-Martinez E.J., Fernandez-de Cordova M.L., Molina-Garcia L., Castilho P.C. (2017). Polyphenolic profile and antioxidant activities of Madeiran elderberry (*Sambucus lanceolata*) as affected by simulated in vitro digestion. Food Res. Int..

[62] Schindler G., Kohlert C., Bischoff R., Maerz R., Ismail C., Veit M., Hahn E., Brinkhaus B. (2001). Pharmacokinetics and bioavailability of an essential oil compound (thymol) after oral administration. Focus Altern. Complement. Ther..

[63] Michiels J., Missotten J., Dierick N., Fremaut D., Maene P., De Smet S. (2008). In vitro degradation and in vivo passage kinetics of carvacrol, thymol, eugenol and trans-cinnamaldehyde along the gastrointestinal tract of piglets. J. Sci. Food Agric..

[64] Li W., Hong B., Li Z., Li Q., Bi K. (2018). GC-MS method for determination and pharmacokinetic study of seven volatile constituents in rat plasma after oral administration of the essential oil of Rhizoma Curcumae. J. Pharm Biomed. Anal..

[65] Mason S.E., Mullen K.A.E., Anderson K.L., Washburn S.P., Yeatts J.L., Baynes R.E. (2017). Pharmacokinetic analysis of thymol, carvacrol and diallyl disulfide after intramammary and topical applications in healthy organic dairy cattle. Food Addit. Contam. Part A-Chem. Anal. Control. Expo. Risk Assess..

[66] Allaoua M., Etienne P., Noirot V., Carayon J.L., Tene N., Bonnafe E., Treilhou M. (2018). Pharmacokinetic and antimicrobial activity of a new carvacrol-based product against a human pathogen, *Campylobacter jejuni*. J. Appl. Microbiol..

[67] Ciganda C., Laborde A. (2003). Herbal infusions used for induced abortion. J. Toxicol. Clin. Toxicol..

[68] Laios K., Lytsikas-Sarlis P., Manes K., Kontaxaki M.I., Karamanou M., Androutsos G. (2019). Drugs for mental illnesses in ancient greek medicine. Psychiatrike.

[69] Woolf A. (1999). Essential oil poisoning. J. Toxicol. Clin. Toxicol..

[70] Franklyne J., Mukherjee A., Chandrasekaran N. (2016). Essential oil micro- and nanoemulsions: Promising roles in antimicrobial therapy targeting human pathogens. Lett. Appl. Microbiol..

[71] Izgi M.N., Telci I., Elmastas M. (2017). Variation in essential oil composition of coriander (*Coriandrum sativum* L.) varieties cultivated in two different ecologies. J. Essent. Oil Res..

[72] Haeseler G., Maue D., Grosskreutz J., Bufler J., Nentwig B., Piepenbrock S., Dengler R., Leuwer M. (2002). Voltage-dependent block of neuronal and skeletal muscle sodium channels by thymol and menthol. Eur. J. Anaesthesiol..

[73] Elmi A., Ventrella D., Barone F., Carnevali G., Filippini G., Pisi A., Benvenuti S., Scozzoli M., Bacci M.L. (2019). In vitro effects of tea tree oil (Melaleuca Alternifolia essential oil) and its principal component terpinen-4-ol on swine spermatozoa. Molecules.

[74] Elmi A., Ventrella D., Barone F., Filippini G., Benvenuti S., Pisi A., Scozzoli M., Bacci M.L. (2017). *Thymbra capitata* (L.) cav. and *Rosmarinus officinalis* (L.) essential oils: In vitro effects and toxicity on swine spermatozoa. Molecules.

[75] Touazi L., Aberkane B., Bellik Y., Moula N., Iguer-Ouada M. (2018). Effect of the essential oil of *Rosmarinus officinalis* (L.) on rooster sperm motility during 4 °C short-term storage. Vet. World.

[76] Ghorbani A., Esmaeilizadeh M. (2017). Pharmacological properties of *Salvia officinalis* and its components. J. Tradit. Complement. Med..

[77] Atsumi T., Fujisawa S., Tonosaki K. (2005). A comparative study of the antioxidant/prooxidant activities of eugenol and isoeugenol with various concentrations and oxidation conditions. Toxicol. Vitr..

[78] Radulovic N.S., Gencic M.S., Stojanovic N.M., Randjelovic P.J., Stojanovic-Radic Z.Z., Stojiljkovic N.I. (2017). Toxic essential oils. Part V: Behaviour modulating and toxic properties of thujones and thujone-containing essential oils of *Salvia officinalis* L., *Artemisia absinthium* L., *Thuja occidentalis* L. and *Tanacetum vulgare* L.. Food Chem. Toxicol..

[79] Mesic A., Mahmutovic-Dizdarevic I., Tahirovic E., Durmisevic I., Eminovic I., Jerkovic-Mujkic A., Besta-Gajevic R. (2019). Evaluation of toxicological and antimicrobial activity of lavender and immortelle essential oils. Drug Chem. Toxicol..

[80] Houdkova M., Doskocil I., Urbanova K., Tulin E.K.C.B., Rondevaldova J., Tulin A.B., Kudera T., Tulin E.E., Zeleny V., Kokoska L. (2018). Evaluation of antipneumonic effect of Philippine essential oils using broth microdilution volatilization method and their lung fibroblasts toxicity. Nat. Prod. Commun..

[81] Houdkova M., Urbanova K., Doskocil I., Rondevaldova J., Novy P., Nguon S., Chrun R., Kokoska L. (2018). In vitro growth-inhibitory effect of cambodian essential oils against pneumonia causing bacteria in liquid and vapour phase and their toxicity to lung fibroblasts. S. Afr. J. Bot..

[82] Andrade M.A., Cardoso M.d.G., Prete P.S.C., Soares M.J., de Azeredo C.M.O., Trento M.V.C., Braga M.A., Marcussi S. (2018). Toxicological aspects of the essential oil from *Cinnamodendron dinisii*. Chem. Biodivers..

[83] Duarte J.A., de Bairros Zambrano L.A., Quintana L.D., Rocha M.B., Schmitt E.G., Boligon A.A., Anraku de Campos M.M., Souza de Oliveira L.F., Machado M.M. (2018). Immunotoxicological evaluation of *Schinus molle* L. (Anacardiaceae) essential oil in lymphocytes and macrophages. Evid.-Based Complement. Altern. Med..

[84] Binder S., Hanakova A., Tomankova K., Pizova K., Bajgar R., Manisova B., Kejlova K., Bendova H., Jirova D., Kolarova H. (2016). Adverse phototoxic effect of essential plant oils on NIH 3T3 cell line after UV light exposure. Cent. Eur. J. Public Health.

[85] Yousefzadi M., Riahi-Madvar A., Hadian J., Rezaee F., Rafiee R. (2012). In vitro cytotoxic and antimicrobial activity of essential oil from *Satureja sahendica*. Toxicol. Environ. Chem..

[86] Borges R.S., Ortiz B.L.S., Pereira A.C.M., Keita H., Carvalho J.C.T. (2019). *Rosmarinus officinalis* essential oil: A review of its phytochemistry, anti-inflammatory activity, and mechanisms of action involved. J. Ethnopharmacol..

[87] Barros F.J., Costa R.J.O., Cesario F., Rodrigues L.B., da Costa J.G.M., Coutinho H.D.M., Galvao H.B.F., de Menezes I.R.A. (2016). Activity of essential oils of *Piper aduncum* anf and *Cinnamomum zeylanicum* by evaluating osmotic and morphologic fragility of erythrocytes. Eur. J. Integr. Med..

[88] Kazemi M. (2015). Chemical composition and antimicrobial, antioxidant activities and anti-inflammatory potential of *Achillea millefolium* L., *Anethum graveolens* L., and *Carum copticum* L. essential oils. J. Herb. Med..

[89] Chikhoune A., Stouvenel L., Iguer-Ouada M., Hazzit M., Schmitt A., Lores P., Wolf J.P., Aissat K., Auger J., Vaiman D. (2015). In-vitro effects of thymus munbyanus essential oil and thymol on human sperm motility and function. Reprod. Biomed. Online.

[90] Yousefzadi M., Riahi-Madvar A., Hadian J., Rezaee F., Rafiee R., Biniaz M. (2014). Toxicity of essential oil of *Satureja khuzistanica*: In vitro cytotoxicity and anti-microbial activity. J. Immunotoxicol..

[91] Habibi E., Shokrzadeh M., Ahmadi A., Chabra A., Naghshvar F., Keshavarz-Maleki R. (2015). Genoprotective effects of *Origanum vulgare* ethanolic extract against cyclophosphamide-induced genotoxicity in mouse bone marrow cells. Pharm. Biol..

[92] Kaur S., Kumar M., Kaur P., Kaur V., Kaur S. (2016). Modulatory effects of *Cassia fistula* fruits against free radicals and genotoxicity of mutagens. Food Chem. Toxicol..

[93] Kalemba-Drozdz M., Cierniak A. (2019). Antioxidant and genoprotective properties of extracts from edible flowers. J. Food Nutr. Res..

[94] Bakkali F., Averbeck S., Averbeck D., Zhiri A., Baudoux D., Idaomar M. (2006). Antigenotoxic effects of three essential oils in diploid yeast (*Saccharomyces cerevisiae*) after treatments with UVC radiation, 8-MOP plus UVA and MMS. Mutat. Res.-Genet. Toxicol. Environ. Mutagenesis.

[95] Hollenbach C.B., Bing R.S., Stedile R., da Silva Mello F.P., Schuch T.L., Alves Rodrigues M.R., de Mello F.B., Braga de Mello J.R. (2015). Reproductive toxicity assessment of *Origanum vulgare* essential oil on male Wistar rats. Acta Sci. Vet..

[96] Fateh A.H., Mohamed Z., Chik Z., Alsalahi A., Zin S.R.M., Alshawsh M.A. (2019). Prenatal developmental toxicity evaluation of *Verbena officinalis* during gestation period in female *Sprague-Dawley* rats. Chem.-Biol. Interact..

[97] Ghadirkhomi A., Safaeian L., Zolfaghari B., Ghazvini M.R.A., Rezaei P. (2016). Evaluation of acute and sub-acute toxicity of *Pinus eldarica* bark extract in Wistar rats. Avicenna J. Phytomed..

[98] Fallahi S., Beyranvand M., Mahmoudvand H., Nayebzadeh H., Kheirandish F., Jahanbakhsh S. (2017). Chemical composition, acute and sub-acute toxicity of *Satureja khuzestanica* essential oil in mice. Marmara Pharm. J..

[99] Lamichhane R., Lee K.-H., Pandeya P.R., Sung K.-K., Lee S., Kim Y.-K., Jung H.-J. (2019). Subcutaneous injection of myrrh essential oil in mice: Acute and subacute toxicity study. Evid.-Based Complement. Altern. Med..

[100] Mishra A.K., Mishra A., Pragya, Chattopadhyay P. (2018). Screening of acute and sub-chronic dermal toxicity of *Calendula officinalis* L essential oil. Regul. Toxicol. Pharmacol..

[101] Daneshbakhsh D., Asgarpanah J., Najafizadeh P., Rastegar T., Mousavi Z. (2018). Safety assessment of *Mentha mozaffarianii* essential oil: Acute and repeated toxicity studies. Iran. J. Med Sci..

[102] Jain N., Sharma M., Joshi S.C., Kaushik U. (2018). Chemical composition, toxicity and antidermatophytic activity of essential oil of *Trachyspermum ammi*. Indian J. Pharm. Sci..

[103] Liaqat I., Riaz N., Saleem Q.-u.-A., Tahir H.M., Arshad M., Arshad N. (2018). Toxicological evaluation of essential oils from some plants of Rutaceae family. Evid.-Based Complement. Altern. Med..

[104] Taghizadeh M., Ostad S.N., Asemi Z., Mahboubi M., Hejazi S., Sharafati-Chaleshtori R., Rashidi A., Akbari H., Sharifi N. (2017). Sub-chronic oral toxicity of *Cuminum cyminum* L.’s essential oil in female wistar rats. Regul. Toxicol. Pharmacol..

[105] Judzentiene A., Garjonyte R. (2016). Compositional variability and toxic activity of Mugwort (*Artemisia vulgaris*) essential oils. Nat. Prod. Commun..

[106] Dahham S.S., Hassan L.E.A., Ahamed M.B.K., Majid A.S.A., Majid A.M.S.A., Zulkepli N.N. (2016). In vivo toxicity and antitumor activity of essential oils extract from agarwood (*Aquilaria crassna*). BMC Complement. Altern. Med..

[107] El-Hosseiny L.S., Alqurashy N.N., Sheweita S.A. (2016). Oxidative stress alleviation by sage essential oil in co-amoxiclav induced hepatotoxicity in rats. Int. J. Biomed. Sci. IJBS.

[108] Aggarwal M.L., Chacko K.M., Kuruvilla B.T. (2016). Systematic and comprehensive investigation of the toxicity of curcuminoid-essential oil complex: A bioavailable turmeric formulation. Mol. Med. Rep..

[109] Hoff Brait D.R., Mattos Vaz M.S., Arrigo J.d.S., Borges de Carvalho L.N., Souza de Araujo F.H., Vani J.M., Mota J.d.S., Lima Cardoso C.A., Oliveira R.J., Negrao F.J. (2015). Toxicological analysis and anti-inflammatory effects of essential oil from *Piper vicosanum* leaves. Regul. Toxicol. Pharmacol..

[110] Mekonnen A., Tesfaye S., Christos S.G., Dires K., Zenebe T., Zegeye N., Shiferaw Y., Lulekal E. (2019). Evaluation of skin irritation and acute and subacute oral toxicity of lavandula angustifolia essential oils in rabbit and mice. J. Toxicol..

[111] Mahmoudvand H., Mahmoudvand H., Oliaee R.T., Kareshk A.T., Mirbadie S.R., Aflatoonian M.R. (2017). In vitro protoscolicidal effects of *Cinnamomum zeylanicum* essential oil and its toxicity in mice. Pharmacogn. Mag..

[112] Llana-Ruiz-Cabello M., Maisanaba S., Puerto M., Pichardo S., Jos A., Moyano R., Camean A.M. (2017). A subchronic 90-day oral toxicity study of *Origanum vulgare* essential oil in rats. Food Chem. Toxicol..

[113] Branquinho L.S., Santos J.A., Lima Cardoso C.A., Mota J.d.S., Lanza Junior U., Leite Kassuya C.A., Arena A.C. (2017). Anti-inflammatory and toxicological evaluation of essential oil from *Piper glabratum* leaves. J. Ethnopharmacol..

[114] Arantes S., Candeias F., Lopes O., Lima M., Pereira M., Tinoco T., Cruz-Morais J., Rosario Martins M.R. (2016). Pharmacological and toxicological studies of essential oil of *Lavandula stoechas* subsp *luisieri*. Planta Med..

[115] Kumar A., Shukla R., Singh P., Dubey N.K. (2010). Chemical composition, antifungal and antiaflatoxigenic activities of *Ocimum sanctum* L. Essential oil and its safety assessment as plant based antimicrobial. Food Chem. Toxicol..

[116] Fandohan P., Gnonlonfin B., Laleye A., Gbenou J.D., Darbouxc R., Moudachirou M. (2008). Toxicity and gastric tolerance of essential oils from *Cymbopogon citratus*, *Ocimum gratissimum* and *Ocimum basilicum* in Wistar rats. Food Chem. Toxicol..

[117] Ribeiro W.L.C., Camurca-Vasconcelos A.L.F., Macedo L.T.F., dos Santos J.M.L., de Araujo J.V., Ribeiro J.D., Pereira V.D., Viana D.D., de Paula H.C.B., Bevilaqua C.M.L. (2015). In vitro effects of *Eucalyptus staigeriana* nanoemulsion on *Haemonchus contortus* and toxicity in rodents. Vet. Parasitol..

[118] Shalaby S.E.M., El-Din M.M., Abo-Donia S.A., Mettwally M., Attia Z.A. (2011). Toxicological affects of essential oils from eucalyptus *Eucalyptus globules* and clove *Eugenia caryophyllus* on albino rats. Pol. J. Environ. Stud..

[119] Ostad S.N., Vazirian M., Pahlevani R., Hadjiakhondi A., Hamedani M.P., Almasian A., Manayi A. (2018). Toxicity evaluation of aromatic water of *Pinus eldarica* Medw. in acute and sub-chronic toxicity experiments. Prog. Nutr..

[120] De Lima R., Guex C.G., da Silva A.R.H., Lhamas C.L., Moreira K.L.D., Casoti R., Dornelles R.C., da Rocha M., da Veiga M.L., Bauermann L.D. (2018). Acute and subacute toxicity and chemical constituents of the hydroethanolic extract of *Verbena litoralis* Kunth. J. Ethnopharmacol..

[121] Kumar R., Sharma R., Patil R.D., Mal G., Kumar A., Patial V., Kumar P., Singh B. (2018). Sub-chronic toxicopathological study of lantadenes of *Lantana camara* weed in Guinea pigs. BMC Vet. Res..

